# Knowledge, Attitudes, and Practices toward Chagas Disease: A Cross-Sectional Survey of Bolivians in the Gran Chaco and Latin American Migrants in London

**DOI:** 10.4269/ajtmh.24-0516

**Published:** 2025-05-06

**Authors:** María del Carmen Díez Hernández, Natalie Elkheir, Temitope Fisayo, Raquel Gonçalves, Edson Grover Sañer Liendo, Caryn Bern, David A. J. Moore

**Affiliations:** ^1^ Médicos del Mundo, Madrid, Spain;; ^2^ London School of Hygiene and Tropical Medicine, London, United Kingdom;; ^3^ Hospital for Tropical Diseases, University College London Hospitals NHS Trust, London, United Kingdom;; ^4^ United Kingdom Chagas Hub, London, United Kingdom;; ^5^ King’s College London, London, United Kingdom;; ^6^ University of Warwick, Coventry, United Kingdom;; ^7^ Hospital Japonés, Santa Cruz de la Sierra, Bolivia;; ^8^ University of California, San Francisco School of Medicine, San Francisco, California

## Abstract

Understanding the at-risk population’s perception of Chagas disease is essential for its effective prevention and control. A cross-sectional survey of knowledge, attitudes, and practices toward Chagas disease was conducted with Bolivians in a highly endemic region of Bolivia and Latin American migrants in London. In total, 175 participants completed the survey: 100 Bolivians in a highly endemic village in Santa Cruz, Bolivia and 75 Latin American migrants in London (of whom 31 were from Bolivia). All participants from the endemic village and all Bolivian migrants in London knew of Chagas disease, whereas only 25% of other Latin American migrants had heard of it (*P* <0.001). In London, Bolivians had more knowledge of Chagas disease than those from other Chagas-endemic countries. In Bolivia only, better understanding of Chagas disease was associated with educational attainment. Only 4% of participants overall were aware of the risk of vertical transmission. Few Latin American migrants in London had previously been tested for Chagas disease, and most were not aware of how to access testing. Migration and level of endemicity may shape individuals’ understanding of as well as attitudes and practices toward Chagas disease. A better understanding of these factors can guide effective prevention and control program development in both endemic and non-endemic settings.

## INTRODUCTION

Chagas disease is a neglected tropical disease endemic (or formerly endemic) in 21 countries of Latin America. It is caused by the parasite *Trypanosoma cruzi *and affects between 6 and 7 million people worldwide.^1^ *T. cruzi* infection in endemic settings is principally transmitted when humans come into contact with the excreta of infected blood-sucking triatomine bugs (vector-borne transmission), which exist throughout the Americas. Because of migration, people with Chagas disease have moved to non-endemic countries, such as those in Europe.^2^^–^^6^ In all settings, *T. cruzi* can be transmitted from mother to child during pregnancy and through blood transfusion and organ transplantation.

Chagas disease has been referred to as a “silent” disease because of its slowly progressing and frequently asymptomatic clinical course. Left undiagnosed and untreated, *T. cruzi* infection can eventually cause cardiac and gastrointestinal complications, typically 20–40 years after initial infection. Long-term cardiac complications include conduction defects, dysrhythmias (a potential cause of sudden cardiac death), and dilated cardiomyopathy. Gastrointestinal involvement may lead to esophageal and colonic dysmotility and eventually, dilatation.^7^ Reactivation in the context of immunosuppression (particularly untreated HIV infection and organ transplantation) can lead to high morbidity and mortality if unrecognized.^8^ Early detection and management of Chagas disease are essential to reduce the risk of vertical transmission, prevent reactivation, and mitigate complications from cardiac and gastrointestinal end-organ disease. This is a particular problem in non-endemic areas where health care professionals often have low awareness of Chagas disease.^9^^–^^11^ Additional barriers to diagnosis in these settings may include a lack of formal screening, testing, and referral pathways. As such, early detection in non-endemic settings depends heavily on people from endemic countries recognizing their risk of Chagas disease and seeking a test. An understanding of the drivers of knowledge, attitudes, and practices (KAP) related to Chagas disease is, therefore, key to appropriate management of infected individuals.

Bolivia has the highest prevalence of Chagas disease in the world.^12^^–^^15^ Chagas disease is increasingly recognized as a public health problem in Europe, home to approximately 4 million documented Latin American migrants.^16^ The prevalence of Chagas disease in this migrant population has been estimated at 4.2%, and it is much higher in some groups (e.g., 18% in Bolivian migrants).^17^ Forty-five percent of cases in Europe are estimated to be in undocumented migrants.^5^ According to the 2021 census, almost 300,000 migrants born in Chagas-endemic countries are now living in the United Kingdom, with approximately half residing in London.^18^

Our primary aim was to explore the knowledge, attitudes, and practices around Chagas disease among the Latin American population in a hyperendemic setting within Bolivia and migrants in a non-endemic setting (the United Kingdom). We sought to identify knowledge gaps and determine any differences between the populations, using this knowledge to formulate strategies for raising awareness of Chagas disease in different settings.

## MATERIALS AND METHODS

### Study design.

We conducted a cross-sectional survey of the Latin American population in two different settings: a hyperendemic village in the Santa Cruz Department of Bolivia and the city of London in the United Kingdom.

### Setting and study population.

In the Bolivian village, all adults currently living in the community were eligible to participate. The study village was located in the Chaco region of Santa Cruz Department, Bolivia in a highly endemic area where the seroprevalence of *T. cruzi* infection was estimated to be 52% overall, with strong age dependence (around 20% in children and rising to 97% in adults older than 30 years of age).^12^^,^^13^ It is an indigenous community that has conserved its Guaraní language and culture. The economy depends on agriculture, cattle, and bakery.^19^ In London, adults who were born or had lived in any of the 21 countries where Chagas disease is endemic (Argentina, Belize, Bolivia, Brazil, Chile, Colombia, Costa Rica, Ecuador, El Salvador, French Guiana, Guatemala, Guyana, Honduras, Mexico, Nicaragua, Panama, Paraguay, Peru, Suriname, Uruguay, and Venezuela) were eligible to participate.

### Sampling.

Participants in both London and Bolivia were recruited through convenience sampling. In Bolivia, participants in the waiting room of the health post were invited to participate. Additionally, community members were invited directly by our researcher visiting residents door to door. In London, an event was organized over two days in a public park where Latin American migrants regularly socialize. Participants were also invited to complete the survey at a shopping center popular with the Latin American migrant community. No formal sample size calculation was performed, and there is no clear consensus on this calculation for KAP studies. However, for quality-of-life-studies (which are similar in format to KAP questionnaire studies), the recommended sample size is 5–10 times the number of items on the questionnaire. Therefore, for the 34-question KAP survey used in this study, the minimum recommended sample was 170 participants.

### Data collection.

Data collection was performed using a structured questionnaire adapted from two previously used questionnaires for suitability in these two settings.^20^^,^^21^ The questionnaire was piloted in both settings. Data collected included sociodemographic characteristics and knowledge, attitudes and behaviors related to the vector, and clinical manifestations and treatment of Chagas disease. All of the surveys were carried out in Spanish. The target population was approached and asked to participate individually. Subjects gave informed consent and were interviewed for approximately 10 minutes.

## STATISTICAL ANALYSES

Sociodemographic characteristics of the population sample are presented using frequency and proportions. The total population of participants was analyzed in three groups: Bolivian people in the endemic village, Bolivian people in London, and non-Bolivian people in London.

To assess associations between knowledge, attitudes, and practices around Chagas disease in these three groups, χ^2^ tests were used. To further evaluate knowledge of Chagas disease, a six-point composite variable (with zero points equating to no knowledge and five points equating to good knowledge) was generated. Participants were given a point each for having heard of Chagas disease, knowledge of the triatomine (vinchuca) vector, knowledge of heart and/or gut involvement, knowledge of treatment availability, and knowledge of at least one method of vector control in houses. Word clouds (with words represented in a size directly proportional to the number of times that they were provided by participants) were generated to visibly display the (up to three) words that participants were asked to provide that they associated with Chagas disease. Attitudes toward Chagas disease were analyzed thematically. Themes were developed deductively as the questionnaire was influenced by theory generated from previous research. A two-tailed *P*-value of <0.05 was considered statistically significant. Data were analyzed with Stata v. 17 (StataCorp, College Station, TX).

## RESULTS

### Participant characteristics.

A total of 175 participants were recruited into the study between June and August 2016: 100 in Bolivia and 75 in London. Table 1 shows the sociodemographic characteristics of study participants.

**Table 1:** Sociodemographic characteristics of study participants in the study village (Bolivia) and in London (United Kingdom)

Characteristics	Study Village in Bolivia, *n* (%)	London, United Kingdom, *n* (%)		All, *n* (%)
		Bolivian	Other Latin American	
Female sex	83 (83)	22 (71)	20 (45)	125 (71)
Median age (years), IQR	33, 24–43	33, 30–42	42, 33–48	36, 26–46
Age group (years)				
18–34	53 (53)	17 (54)	12 (27)	82 (47)
35–54	32 (32)	12 (39)	26 (50)	70 (40)
55–73	15 (15)	2 (6.4)	6 (14)	23 (13)
Education				
Primary or less	38 (38)	7 (23)	4 (9)	49 (28)
Secondary/high school	48 (48)	15 (48)	27 (61)	90 (51)
Technical school or university	14 (14)	9 (29)	13 (30)	36 (21)
Occupation				
Homemaker	57 (57)	6 (19)	3 (7)	66 (38)
Student	7 (7)	1 (3)	0	8 (5)
Worker	36 (36)	23 (74)	40 (90)	99 (57)
Unemployed	0	1 (3)	0	1 (1)
Retired	0	0	1 (2)	1 (1)
Family member with Chagas disease	79 (79)	13 (42)	0 (0)	97 (67)
Self-reported having seen a triatomine insect	100 (100)	27 (87)	4 (9)	129 (74)
Total	100	31	44	175

IQR = interquartile range.

In Bolivia, 89% of participants were born in the same province as the study village, whereas 11 participants were born in other provinces or departments. Fifty-seven percent of participants in Bolivia were homemakers (all women); 7% were students; and 36% were employed in agriculture, food selling, or cleaning.

In London, 31 participants were from Bolivia, and 44 were from other endemic countries where Chagas disease is less prevalent (27 participants from Ecuador, 13 participants from Colombia, two participants from Venezuela, one participant from Nicaragua, and one participant from Peru). In London, the majority of participants were employed (74.2% among Bolivian people and 90.9% of non-Bolivian migrants), and the majority worked as cleaners, with some participants working in the catering industry as waiters or chefs.

### Knowledge of Chagas disease.

All participants in the study village had heard of Chagas disease as had all Bolivians in London, whereas only 11 of 44 (25%) migrants from other (lower-endemicity) countries had heard of it. Seventy-seven percent of participants in the study village knew that Chagas disease was transmitted by the triatomine vector versus 81% of Bolivian migrants in London and just 9% of non-Bolivian migrants in London. Notably, only seven participants were aware of vertical transmission: three participants from the study village (3%) and four Bolivian migrants in London (2%). Table 2 shows the proportion of people surveyed in Bolivia and London with any knowledge of Chagas disease relating to its transmission, clinical features, treatment, and vectorial control plus a combined composite point-score variable (from zero to five, with zero equating to no knowledge of Chagas disease and five equating to good knowledge). Overall knowledge of participants in the study village was very similar to that of Bolivian migrants in London.

**Table 2:** Knowledge of Chagas disease of study participants in the study village (Bolivia) and in London (United Kingdom)

Knowledge	Study Village in Bolivia (*n* = 100), *n* (%)	London, United Kingdom, *n* (%, 95% CI)	
		Bolivian (*n* = 31)	Other Latin American (*n* = 44)
Heard of Chagas disease	100 (100, 96–100)	31 (100, 89–100)	11 (25, 13–40)
Transmission—knew triatomine	80 (80, 71–87)	25 (81, 63–93)	4 (9, 3–22)
Aware that it can affect heart and/or gut	55 (55, 45–65)	15 (48, 30–67)	1 (2, 0–12)
Knew medication available to treat	35 (35, 26–45)	17 (55, 36–73)	2 (5, 0–15)
Knew at least one method of vector control in the house	97 (97, 91–99)	29 (94, 79–99)	5 (11, 4–25)
Knowledge score*			
0	0 (0)	0 (0)	33 (75)
1	1 (1)	0 (0)	6 (14)
2	10 (10)	5 (16)	1 (2)
3	28 (28)	5 (16)	2 (5)
4	43 (43)	13 (42)	1 (2)
5	18 (18)	8 (26)	1 (2)

* Participants were given one point each for 1) having heard of Chagas disease, 2) awareness of the triatomine vector, 3) awareness of heart and/or gut involvement, 4) knowledge of treatment availability, and 5) knowledge of at least one method of vector control in houses.

### Attitudes toward Chagas disease.

In the study village, 77% of participants perceived Chagas disease to be a serious problem in their community versus 57% of Bolivians in London (*P* <0.05). None of the non-Bolivians in London who had heard of Chagas disease perceived it to be a serious problem. Seventy-three percent of participants in Bolivia thought that Chagas disease could be cured compared with 53% of Bolivians in London (*P* <0.001). Participants in Bolivia were more likely to report that Chagas disease could be treated using herbs or diets than Bolivians in London (24% versus 10%, *P* <0.05). Only seven of the non-Bolivian respondents answered the question on whether Chagas disease can be cured; six of them (86%) thought that it could be.

**Figure 1.**
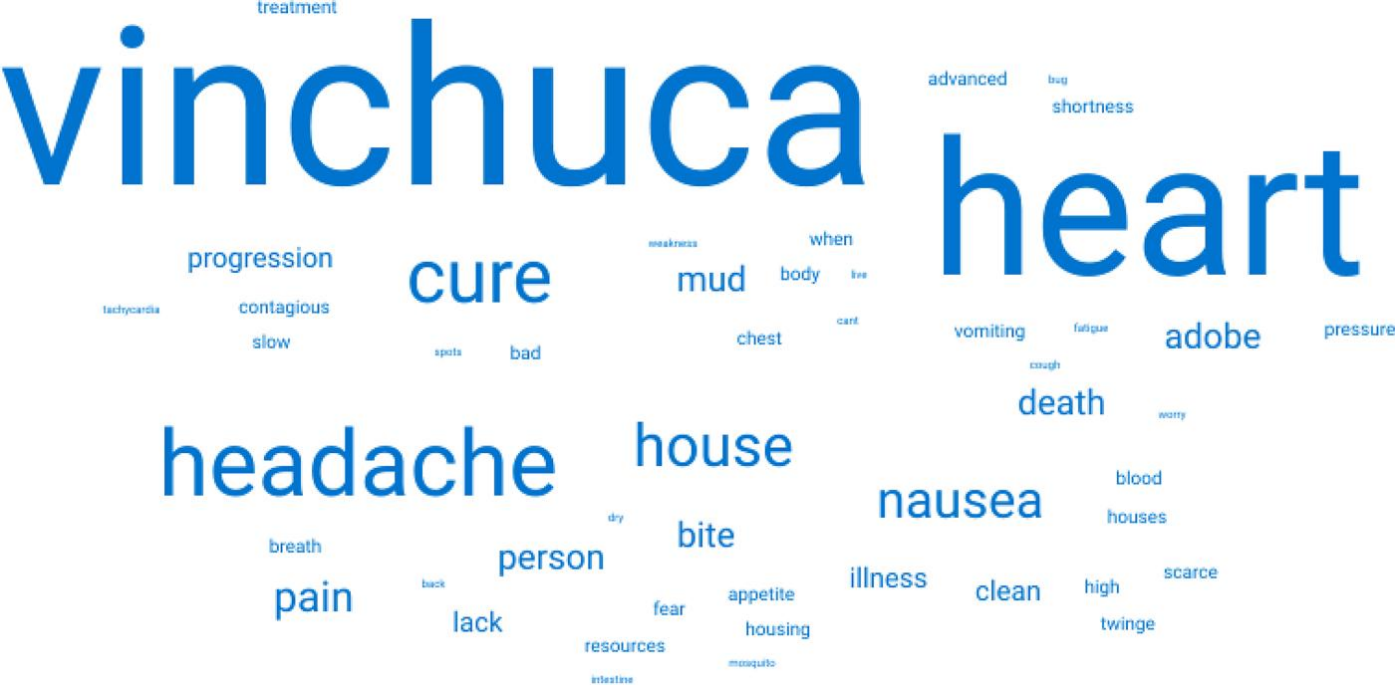
Word cloud from the word association with Chagas disease from participants in the study village in Bolivia.

**Figure 2.**
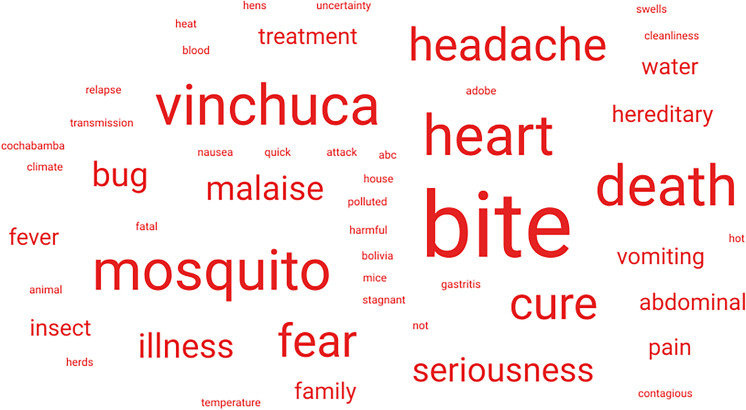
Word cloud from the word association with Chagas disease from 75 Latin Americans (both Bolivians and non-Bolivians) living in London, United Kingdom.

Participants who had heard of Chagas disease were asked to give three words that they associate with Chagas disease. Figures 1 and 2 are word clouds of the received responses from Bolivians in Bolivia and Latin American migrants in London, respectively. In total, 102 participants (66 in Bolivia and 36 in London) provided at least one word. A total of 233 words (of which 90 were unique) were provided in total. Among the participants in Bolivia, the three most popular words were “vinchuca” (a colloquial term for the triatomine vector), “heart,” and “headache,” accounting for 26, 16, and 11 responses, respectively. In London, the most common word was “bite(s)” with eight responses followed by “vinchuca” and “mosquito” with five responses each. Across both groups, Chagas disease evoked responses such as “fear,” “death,” and “no cure.” Words related to the body and to symptoms, such as “pain,” “fatigue,” and “dizziness,” were also in both samples.

### Practices around Chagas disease.

Participants in Bolivia and London differed regarding their practices toward Chagas disease testing and treatment (Table 3). Seventy-nine percent of the participants from the study village reported having previously been tested versus 55% of Bolivians in London and just 5% of non-Bolivians in London (*P* <0.001). Participants in Bolivia reported that they would visit a local health post (57%) or public hospital (25%) if they suspected Chagas disease, and 16% would not seek medical care. In London, Bolivian migrants would mostly visit a public hospital (58%) followed by local primary care center (32%), and one participant reported that they would seek a private doctor. Of the 11 non-Bolivian respondents, 6 (55%) would go to a local primary care center versus 5 (45%) would go to a public hospital.

**Table 3:** Practices around self-reported testing and treatment of Chagas disease in Bolivians in the study village, Bolivians living in London, and other Latin American migrants living in London

Practices	Study Village in Bolivia (*n* = 100), *n* (%)	London, United Kingdom, *n* (%)		*P*-Value*
		Bolivian (*n* = 31)	Other Latin American (*n* = 44)	
Previously tested for Chagas disease	79 (79)	17 (55)	2 (5)	<0.001
Reported test result				
Positive	46 (58)	3 (18)	0 (0)	<0.05
Negative	30 (38)	12 (71)	0 (0)	
Do not know	3 (0.4)	2 (12)	0 (0)	
Reported previous treatment received	10 (21.7)	2 (75)	0 (0)	–
Mean reported duration of treatment	2.7	2	–	–
Where would you go if you suspected Chagas disease?				
Public hospital	25 (25)	18 (58)	5 (11)	<0.01
Private hospital	0 (0)	0 (0)	0 (0)	
Health center	57 (57)	10 (32)	6 (14)	
Private doctor	0 (0)	1 (3)	0 (0)	
Traditional healer	0 (0)	0 (0)	0 (0)	
Would not seek care	16 (16)	0 (0)	0 (0)	
I do not know	2 (2)	2 (7)	0 (0)	

* *P*-value refers to the χ^2^ test performed for association between self-reported testing and treatment proportions and group (Bolivians in the study village, Bolivians in London, or other Latin Americans in London).

## DISCUSSION

This study explored and compared knowledge, attitudes, and practices around Chagas disease between a community living in a hyperendemic region of Bolivia and migrants born in endemic countries and living in London. We found that Bolivian participants (in both Bolivia and London) had a greater level of knowledge about the transmission, clinical features, and treatment of Chagas disease than non-Bolivian participants. Awareness of vertical transmission was universally low. Few Bolivians in London perceived Chagas disease to be a serious risk to them, which reflects reality for those from non-endemic regions, but it may suggest a misconception for those from endemic regions who think that they are no longer at risk after emigrating (overlooking the asymptomatic indeterminate phase of the disease). Participants in Bolivia were more likely to have been tested for Chagas disease than those in London and were more likely to have had a positive result.

Our findings add to the previous body of evidence on populations’ understanding of Chagas disease in both endemic and non-endemic settings. Previous literature from endemic settings describes striking inconsistencies in knowledge. For example, one study of 146 pregnant women in the Ecuadorian Amazon found that 16% of participants had some knowledge of Chagas disease.^22^ By contrast, the majority could recognize the triatomine vector. That some people living in Chagas-endemic areas are unable to draw a link between the triatomine bug and Chagas disease (which was the case for 13% of our participants in the study village) is a finding that has been replicated by studies in Argentina and Mexico, suggesting a wider problem throughout Latin America.^23^^,^^24^ In our study, knowledge of vector control measures was reasonably good among the Bolivian population, whereas the clinical features and treatment were poorly understood. This could suggest some success of local efforts, which have focused primarily on vector control and raising awareness in the context of program activities. However, further improvements in educational programs could include supporting better understanding of the clinical features of Chagas disease, which may aid diagnosis. Furthermore, educational programs should address beliefs around the value of antiparasitic therapy early in the course of infection. Twenty-four percent of participants in Bolivia reported that Chagas disease could be treated using medicinal herbs (compared with 10% of Bolivians in London).

Of our study village participants who reported previous testing for Chagas, 58% reported a previous positive result, and 22% had received treatment. This may reflect national and local management policies, which do not include universal antiparasitic treatment of adults; however, it was beyond the scope of this study to investigate this diagnosis to treatment gap.

Our study also adds insight to knowledge, attitudes, and practices of Chagas disease among at-risk migrants living in non-endemic settings. Some previous research suggests that knowledge about Chagas disease is generally low in non-endemic settings. For example, a German survey of 43 Bolivian migrants found that over half had no knowledge regarding symptoms of Chagas disease, and a third could not identify routes of transmission.^25^ Our study differed in that we found that overall knowledge of Chagas disease was higher in migrants from Bolivia than those from other lower-endemicity countries in Latin America, similar to previous research in Italy.^10^ This finding stands to reason; if a disease is not common in your country of origin, you may not know about it, whereas if over half of your community is affected, you will. This is further supported by our finding that Bolivian migrants who knew a friend or family member with Chagas disease had greater knowledge of the disease, suggesting the importance of knowledge dissemination through informal routes. Additionally, having a relative with known Chagas disease may also reflect higher prevalence in that person’s community of origin. Failure of participants in our study to recognize specific routes of transmission, in particular vertical transmission, has also been described among Bolivian migrants in Japan.^26^

Because of the possibility of *T. cruzi* infection in childhood, the Bolivian migrants in our study may have a high prevalence of Chagas disease (early findings from a community screening initiative of Chagas disease in Latin American migrants in London detected a prevalence of 23%); however, Bolivian migrants in London perceived their risk to be very low.^27^ To address this gap between perception and actual risk, educational programs in non-endemic settings must highlight the asymptomatic indeterminate phase of Chagas disease, that migration does not eliminate one’s risk, and the ongoing risk of vertical transmission.

Testing migrants for *T. cruzi *infection in non-endemic settings is usually reliant on self-reporting (awareness of one’s risk) and/or indiscriminate screening approaches targeting migrants from the whole Latin America region (as we do not have suitable risk stratification or targeted approaches). For example, the Migrant Health Guide published by the United Kingdom Health Security Agency recommends screening all women of reproductive age or pregnant women from the 21 endemic countries.^4^ Given that migrants from this whole Latin America region (which will include a sizable proportion of people at no or very low risk) will be offered screening tests under such policies in non-endemic settings, understanding of their knowledge, attitudes, and perceptions is very helpful information to feed into non-endemic setting screening policy and program implementation. The issue of underdiagnosis of Chagas disease among at-risk migrants has been researched in the United States, where understanding physicians’ knowledge, attitudes, and practices toward Chagas disease has supported the development of educational interventions that have successfully resulted in increased testing for Chagas disease.^28^

Interestingly, migrants in London reported that they were more likely to attend a hospital rather than primary care facility if they suspected Chagas disease. This suggests some misunderstanding about how to navigate the health care system as in the United Kingdom, primary health care facilities are the gateway to the National Health Service. This finding supports previous research that has suggested low registration with primary care services among Latin American migrants in London.^29^ In non-endemic settings, engagement and education of Latin American communities are needed to raise awareness of their rights to access (and how to navigate) primary health care and testing for Chagas disease.

The strengths of this study lie in its novel design and the richness of data captured by the questionnaire, allowing us to make comparisons and recommendations about Chagas disease across endemic and non-endemic settings. Further, our methodology has allowed for a more in-depth analysis of the drivers of Chagas disease understanding and behaviors than previous research has allowed. However, this study is not without limitations. First, our (convenience) sampling strategy did not result in a representative study population, and men were particularly underrepresented in the study village. Of those who reported having been tested for Chagas disease, 58% in the study village reported a positive test. This is slightly lower than the expected age-specific prevalence, which could suggest that the study population differs from the wider population in that they have a lower disease prevalence. In London, the absence of age-specific prevalence estimates in migrants hinders any ability to quantify how representative the sample is likely to be based on Chagas disease prevalence. Another limitation of this study is that there were some themes identified from the questionnaire around perceptions of risk and health care-seeking behaviors that would require qualitative methods to explore further, and we recommend future research to address these issues. Furthermore, we were not able to accurately assess participants’ actual risk of Chagas disease, which limited our interpretation of any gap between perceived and actual risk. It should also be noted that during the data collection period of this study, the study community in Bolivia was actively participating in different activities related to triatomine vector control, including assessment of indoor residual spraying practices and housing improvement.^13^^,^^30^^,^^31^ Because of the close contact that vector control researchers had with the study village during the conduct of our study, some bias may have been introduced into the survey responses. Additionally, as participants were selected from waiting areas of health posts, they may have been more health literate or have received more health education than the wider population. A further limitation of this study is the lag between data collection and publication of results as it is possible that some findings may have changed. Finally, because of language constraints, those in the study village who did not speak Spanish were excluded, which may have introduced some bias (for example, older people were more likely to speak only the indigenous Guaraní language).

## CONCLUSION

Migration and level of endemicity may shape individuals’ understanding of as well as attitudes and practices around Chagas disease. A better understanding of these factors could help to guide effective prevention and control programs in both endemic and non-endemic settings.

## References

[1] World Health Organization, 2023. Chagas Disease (Also Known as American trypanosomiasis). Available at: https://www.who.int/news-room/fact-sheets/detail/chagas-disease-(american-trypanosomiasis). Accessed December 20, 2024.

[2] Coura JR Viñas PA Junqueira AC, 2014. Ecoepidemiology, short history and control of Chagas disease in the endemic countries and the new challenge for non-endemic countries. Mem Inst Oswaldo Cruz 109: 856–862. 25410988 10.1590/0074-0276140236 PMC4296489

[3] World Health Organization, 2009. Control and Prevention of Chagas Disease in Europe. Report of a WHO Informal Consultation, Geneva. Available at: https://www.who.int/publications/i/item/WHO-HTM-NTD-IDM-2010.1. Accessed June 26, 2025.

[4] United Kingdom Government Office for Health Improvement and Disparities, 2021. Chagas Disease: Migrant Health Guide. Advice and Guidance on the Health Needs of Migrant Patients for Healthcare Practitioners. Available at: https://www.gov.uk/guidance/chagas-disease-migrant-health-guide. Accessed June 26, 2025.

[5] Basile L ; Working Group on Chagas Disease Collective, 2011. Chagas disease in European countries: The challenge of a surveillance system. Eurosurveillance 16: 19968. 21944556

[6] Gascon J Bern C Pinazo MJ, 2010. Chagas disease in Spain, the United States and other non-endemic countries. Acta Trop 115: 22–27. 19646412 10.1016/j.actatropica.2009.07.019

[7] Pérez-Molina JA Molina I, 2018. Chagas disease. Lancet 391: 82–94. 28673423 10.1016/S0140-6736(17)31612-4

[8] Lattes R Lasala MB, 2014. Chagas disease in the immunosuppressed patient. Clin Microbiol Infect 20: 300–309. 24602129 10.1111/1469-0691.12585

[9] Blasco-Hernández T García-San Miguel L Navaza B Navarro M Benito A, 2016. Knowledge and experiences of Chagas disease in Bolivian women living in Spain: A qualitative study. Glob Health Action 9: 30201. 26976265 10.3402/gha.v9.30201 PMC4789531

[10] Di Girolamo C , 2016. Chagas disease in a non-endemic country: A multidisciplinary research, Bologna, Italy. J Immigr Minor Health 18: 616–623. 25935443 10.1007/s10903-015-0214-0

[11] Iglesias-Rus L Romay-Barja M Boquete T Benito A Blasco-Hernández T, 2019. The role of the first level of health care in the approach to Chagas disease in a non-endemic country. PLoS Negl Trop Dis 13: e0007937. 31841503 10.1371/journal.pntd.0007937 PMC6913928

[12] Samuels AM ; Working Group on Chagas Disease in Bolivia and Peru, 2013. Epidemiology of and impact of insecticide spraying on Chagas disease in communities in the Bolivian Chaco. *PLoS Negl Trop Dis* 7: e2358. 23936581 10.1371/journal.pntd.0002358 PMC3731239

[13] Hopkins T Gonçalves R Mamani J Courtenay O Bern C, 2019. Chagas disease in the Bolivian Chaco: Persistent transmission indicated by childhood seroscreening study. Int J Infect Dis 86: 175–177. 31357060 10.1016/j.ijid.2019.07.020

[14] Verdú J Ruiz MT, 2003. Control of Chagas’ disease in Guarani communities: Knowledge and hygiene habits within the Project to Improve Living Conditions in Bolivia. Gac Sanit 17: 166–168. 12729546 10.1016/s0213-9111(03)71717-8

[15] Forsyth C, 2015. Controlled but not cured: Structural processes and explanatory models of Chagas disease in tropical Bolivia. Soc Sci Med 145: 7–16. 26432176 10.1016/j.socscimed.2015.09.022

[16] Román-Velázquez P Retis J, 2021. Narratives of Migration, Relocation and Belonging: Latin Americans in London. Cham, Switzerland: Palgrave Macmillan.

[17] Requena-Méndez A Aldasoro E de Lazzari E Sicuri E Brown M Moore DA Gascon J Muñoz J, 2015. Prevalence of Chagas disease in Latin-American migrants living in Europe: A systematic review and meta-analysis. PLoS Negl Trop Dis 9: e0003540. 25680190 10.1371/journal.pntd.0003540 PMC4332678

[18] Office for National Statistics, 2021. Census 2021. Available at: https://www.nomisweb.co.uk/sources/census_2021. Accessed June 26, 2025.

[19] Vision Internacional Bolivia, 2015. PERIÓDICO VISION"Z" DE Santa Cruz. Estado Plurinacional de Bolivia. Ejercemos un periodismo comprometido con la verdad, la justicia y la razón. LA VERDAD SIEMPRE ES REVOLUCIONARIA. Available at: https://visionzinternacionalbolivia.blogspot.com/2016/06/comunidad-indigena-guarani-itanambikua.htm. Accessed December 20, 2024.

[20] Donovan SD Stevens M Sanogo K Masroor N Bearman G, 2014. Knowledge and perceptions of Chagas disease in a rural Honduran community. Rural Remote Health 14: 2845. 25204581

[21] Sanchez DR Traina MI Hernandez S Smer AM Khamag H Meymandi SK, 2014. Chagas disease awareness among Latin American immigrants living in Los Angeles, California. Am J Trop Med Hyg 91: 915–919. 25200261 10.4269/ajtmh.14-0305 PMC4228887

[22] Restrepo Zambrano M Rouset F Carrasco OF Echeverría Murillo D Costales JA Brenière SF, 2019. Congenital Chagas disease in the Ecuadorian Amazon: Maternal screening at delivery and evaluation of risk factors associated with vector exposure. Am J Trop Med Hyg 101: 1350–1358. 31595866 10.4269/ajtmh.19-0340 PMC6896853

[23] Valdez-Tah A Huicochea-Gómez L Ortega-Canto J Nazar-Beutelspacher A Ramsey JM, 2015. Social representations and practices towards triatomines and Chagas disease in Calakmul, México. PLoS One 10: e0132830. 26204555 10.1371/journal.pone.0132830 PMC4512683

[24] Llovet I Dinardi G Canevari C Torabi N, 2016. Health care seeking behavior of persons with acute Chagas disease in rural Argentina: A qualitative view. J Trop Med 2016: 4561951. 27829843 10.1155/2016/4561951 PMC5088329

[25] Navarro M , 2017. Cross-sectional, descriptive study of Chagas disease among citizens of Bolivian origin living in Munich, Germany. BMJ Open 7: e013960. 10.1136/bmjopen-2016-013960 PMC5253600 28093440

[26] Iglesias Rodríguez IM Mizukami S Manh DH Thuan TM Justiniano HA Miura S Ito G Huy NT Smith C Hirayama K, 2020. Knowledge, behaviour and attitudes towards Chagas disease among the Bolivian migrant population living in Japan: A cross-sectional study. BMJ Open 10: e032546. 10.1136/bmjopen-2019-032546 PMC7490920 32928842

[27] Elkheir N Dominic C, 2022. One in Five Positive: Community Screening for Chagas Disease in London, UK. American Society of Tropical Medicine and Hygiene Conference 2022, October 30 to November 3, 2022: Seattle, WA.

[28] Wheelock A Sandhu S Reifler K Carrion M Bourque DL Hamer DH Hochberg NS, 2024. Improving clinician awareness of and screening for Chagas disease with an educational intervention. Am J Trop Med Hyg 111: 380–386. 38889732 10.4269/ajtmh.23-0483 PMC11310624

[29] McIlwaine C Bunge D, 2016. Towards Visibility: The Latin American Community in London. Available at: https://trustforlondon.org.uk/research/towards-visibility-latin-american-community-london. Accessed December 20, 2024.

[30] Gonçalves R Landivar D Grover Sañez Liendo E Mamani Fernandez J Ismail HM Paine MJI Courtenay O Bern C, 2021. Improving houses in the Bolivian Chaco increases effectiveness of residual insecticide spraying against infestation with Triatoma infestans, vector of Chagas disease. Trop Med Int Health 26: 1127–1138. 34114721 10.1111/tmi.13640

[31] Gonçalves R Logan RAE Ismail HM Paine MJI Bern C Courtenay O, 2021. Indoor residual spraying practices against Triatoma infestans in the Bolivian Chaco: Contributing factors to suboptimal insecticide delivery to treated households. Parasit Vectors 14: 327. 34134775 10.1186/s13071-021-04831-1 PMC8207695

